# Involving supernumerary teeth in “qpdb” teeth numbering system

**DOI:** 10.1038/s41598-026-58563-2

**Published:** 2026-07-03

**Authors:** Rami Rabie Shehabeldin, Aya Maher Elgamal, Kaber Osama Khafagy, Mohamed Ehab Embaby, Mahitabe Elgamily

**Affiliations:** 1 Oral Biology Department, Faculty of Dentistry, Mansoura University, Mansoura, Egypt 35516; 2 Oral Biology Department, Faculty of Oral and Dental Medicine, Alsalam University, Tanta, Egypt 31511; 3 Faculty of Oral and Dental Medicine, Alsalam University, Tanta, Egypt

**Keywords:** Supernumerary teeth, Teeth numbering system, qpdb system, Palmer notation, Universal, Health care, Medical research

## Abstract

Supernumerary teeth are extra-dentation or tooth-like structure that may be present anywhere in the oral cavity. Currently used numbering systems lack a standardized method for identifying supernumerary teeth which might result in misinterpretation and hinder effective collaboration among dental practitioners. A pre-study questionnaire was conducted on 235 dental practitioners, including dentists, interns and clinical dental students to evaluate their previous experience with supernumerary teeth and the numbering systems used to identify them. Of the respondents, only 32% reported using systems to identify supernumeraries while 68% had no experience with any of systems confirming a significant gap in standardized practice highlighting the need for development of a new numbering system. This manuscript presents a novel addition to the existing “qpdb” tooth numbering system for identification of supernumerary teeth, addressing the limitations of existing methods that fail to provide a standardized approach for identifying these extra teeth. The modification introduces the digit zero (0) placed in combination with the tooth number mesially adjacent to the supernumerary (the identifier tooth) as a designation. This approach enables more precise, consistent and communicable identification of supernumerary teeth addressing the limitations of currently available numbering systems.

**Supplementary Information:**

The online version contains supplementary material available at 10.1038/s41598-026-58563-2.

## Introduction

Supernumerary teeth (SNT) are defined as any tooth or tooth-like structure that exceeds the normal complement of primary and permanent dentition located in the oral cavity^1^. The prevalence of supernumerary teeth varies between 0.1% and 3.8%, with higher frequency observed in permanent dentition^2–4^. Furthermore, there is a gender predilection in males to females by ratio 2:1^5^.

The etiology of supernumerary teeth is still unknown^6^, however, it is often attributed to various factors such as genetic factors, environmental influences and developmental issues^7^. Previous studies suggested multiple theories to clarify the etiology, such as atavism, dichotomy, embryonic aberrations theory and hyperactivity of the dental lamina, which is the most generally accepted^8^. Hereditary theory is another theory which admitted genetic factor as important contributor for formation of extra-odontomas^8–10^.

Supernumerary teeth are present in various configurations either solitary or as multiples, unilaterally or bilaterally and it also might be observed in either or both Jaws^11–14^. Morphologically, they are categorized as conical, tuberculate, molari-form, supplemental or odontomes^15–17^. Additionally, a location-based classification system identified four main types: mesiodens, parapremolars, paramolars, and distomolars^18–36^.

Clinically supernumerary teeth may cause a range of complications including failure of eruption and retention of primary and permanent dentition^37^. Consequently, displacement, misalignment, and rotation of teeth might be observed, all of which carry significant implications for diagnosis and treatment planning^38^. Effective management of supernumerary teeth depends on accurate identification, documentation and communication among dental practitioners. In current clinical practice three common systems are predominantly employed for the identification of deciduous and permanent dentition: universal, Palmer/Zsigmondy & and the Fédération Dentaire Internationale (FDI) system^38^. Despite of their widespread adoption, none of these systems provides a standardized universally agreed method for precise identification and classification of supernumerary teeth which might result in misinterpretation and delay the communication among dental practitioners and complicates the interaction in multidisciplinary setting^39,40^.

To address this clinical gap, dentists seek a dependable numbering system that accurately associates specific details with each tooth (38). A recently introduced “qpdb” numbering system, divides the oral cavity into four quadrants, each designated by a letter whose morphology reflects its anatomical position relative to the midline, this system aims to streamline dental charting (39). The straight line in each letter represent the midline and the head of the letter represented the selected quadrant. So the (q) represented the maxillary right, (p) for the maxillary left, (d) for the mandibular right & (b) for the mandibular left quadrant. The permanent teeth are represented in numbers from 1 to 8 in each quadrant starting from central incisor, while in the primary teeth capital letters were used instead of numbers, (A, B, C, D & E) starting from central incisor ending with the second deciduous molar^39^.

Although the qpdb system offers a streamlined and visually intuitive approach to dental charting, its current version does not incorporate a systematic method for the identification of supernumerary teeth, representing a limitation. Consequently, the aim of this study was to overcome this limitation by proposing a structured modification of the qpdb numbering system that enables standardized identification and documentation of supernumerary teeth. A pre-study questionnaire was conducted among dental practitioners to confirm the clinical need for such a modification prior to its development.

## Methodology

Ethical approval was granted by the research ethics committee of Faculty of Oral and Dental Medicine Alsalam University, Egypt with code no (Sue010401264). The study was conducted in compliance with the principles of declaration of Helsinki.

### Questionnaire design and validation

A structured pre-study questionnaire was specifically developed for this study (supplementary file1). It was designed to prioritize, clarity of purpose, ease of completion, and complete anonymity with no personal identifiers collected at any stage. Participation was entirely voluntary, participants were informed that the questionnaire is for research work and that it will be published. All participants provided informed consents prior to participation; consents were embedded within the questionnaire instrument itself. Individuals who declined to provide consent were under no obligation to complete the questionnaire.

The content validity of the questionnaire was established through review by a panel of experienced dental clinicians prior to distribution and minor revisions were made based on their feedback to ensure that all items were clearly worded and relevant to the objectives.

### Sampling strategy and participants

Participants were recruited using convenience sampling through online digital platforms, including professional dental networks and academic institutional channels, to ensure wide reach and efficient data collection.

#### Inclusion criteria:

licensed practicing dentists, intern dentists completing their clinical training year, and final year clinical dental students actively engaged in patient care.

#### Exclusion criteria:

non clinical dental staff, preclinical students with no direct patient exposure, and participants who did not complete the questionnaire in full.

A total of 235 dental practitioners were enrolled, comprising dentists, interns and clinical dental students, the questionnaire collected both qualitative and quantitative data across the following domains: prior clinical experience with supernumerary teeth; frequency of encountered cases; the most commonly encountered supernumerary tooth; familiarity with existing tooth numbering systems used to identify supernumerary teeth and the methods of communication currently employed to describe such teeth in clinical practice.

### Statistical analysis

Data collected from the questionnaire were coded and analyzed using SPSS (Statistical Package for the Social Sciences, version 26.0; IBM Corp., Armonk, NY, USA). Descriptive statistics were computed, presenting categorical variables as frequencies and percentages. To evaluate the associations between the participants’ professional level (clinical students, interns, and practicing dentists) and their clinical encounters or system numbering preferences, the Pearson Chi-Square test was implemented.

## Results

### Outcomes of the questionnaire and their statistical implementation

**Fig. 1:**
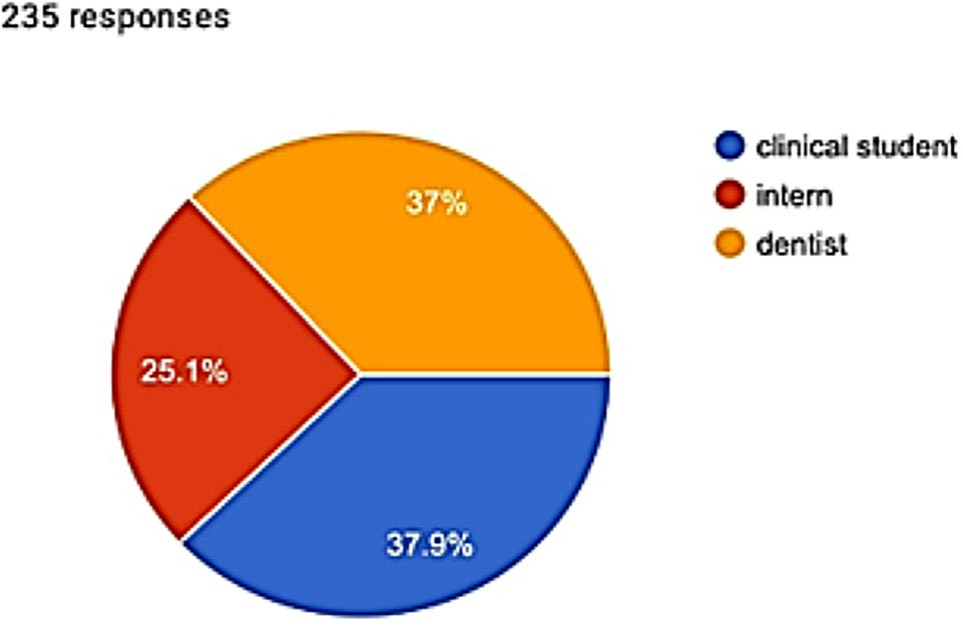
Distribution of participants by professional status.

**Fig. 2:**
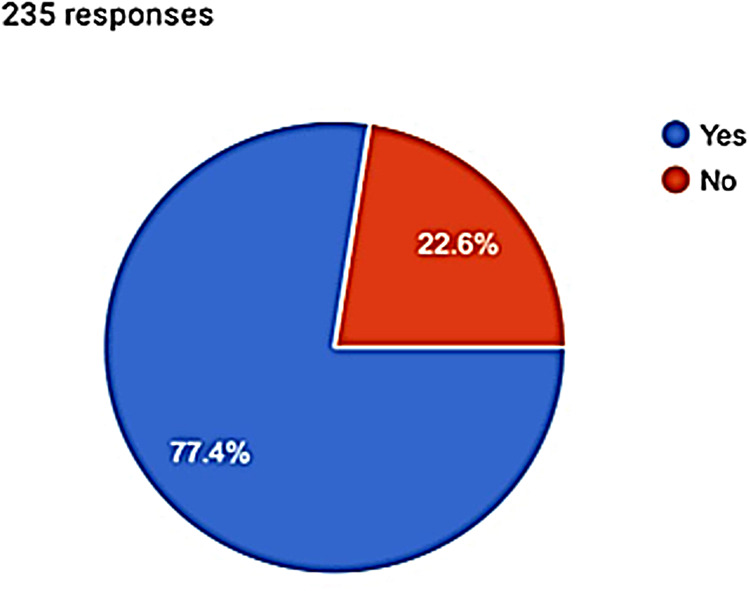
Clinical exposure to supernumerary teeth among.

**Fig. 3:**
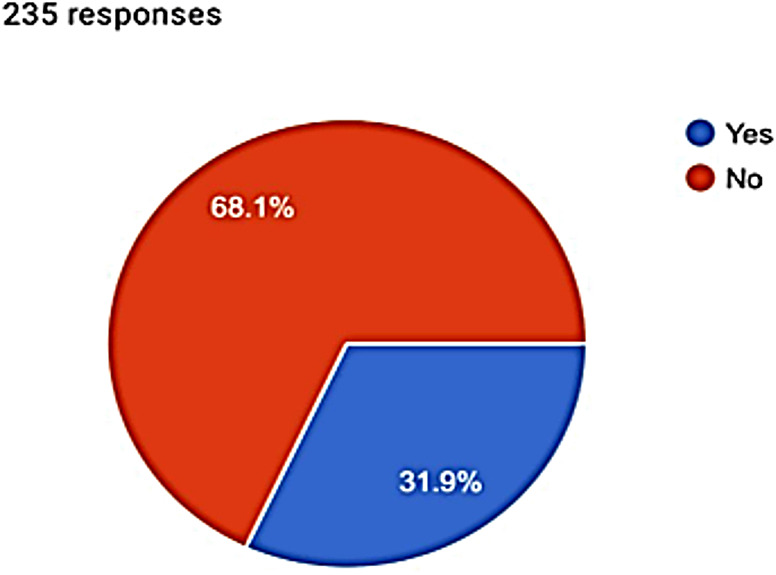
Participants’ experience with numbering systems for supernumerary teeth.

The outcomes of the questionnaire were summarized in (Figs. 1, 2 and 3). The questionnaire was completed by 235 participants, of whom 38% were dental students, 37% were dentists and 25% interns (Fig. 1).

77.4% of respondents had encountered supernumerary teeth during their clinical work, while 22.6% reported having no prior experience managing such cases (Fig. 2).

Regarding the use of numbering systems to indicate supernumeraries, only 32% of participants reported using such systems to identify them. On the other hand, the majority 68% had no experience with any of systems, which highlights the need for a straightforward system to indicate such teeth in an accessible manner (Fig. 3).

### Clinical encounters

A Pearson Chi-Square test (Table 1) was conducted to examine the relationship between the participants’ professional level and their clinical exposure to supernumerary teeth. The analysis revealed a highly statistically significant association (*p* < 0.001). Practicing dentists demonstrated a profoundly higher rate of clinical encounters (93.1%) compared to interns (79.7%) and clinical students (60.7%), indicating that the clinical necessity for managing and naming these anomalies increases fundamentally with advanced clinical experience.

**Table 1:** Chi-Square analysis of clinical encounters with supernumerary teeth across different professions.

Professions	Encountered supernumerary teeth? n (%)		p-value
	Yes	No	
Clinical student	54 (60.7%)	35 (39.3%)	< 0.001*
Intern	47 (79.7%)	12 (20.3%)	
Dentist	81 (93.1%)	6 (6.9%)	
Total	182	53	

### Utilization of numbering systems

A Pearson Chi-Square test (Table 2) revealed that there is no statistically significant association between the participants’ professional level and their use of a tooth numbering system (*p* = 0.057). This lack of statistical significance indicates that the absence of utilizing a standardized naming methodology is a widespread, universal issue across the dental profession, regardless of clinical experience. Even among practicing dentists who routinely encounter these anomalies, the majority (60.9%) do not implement a specific numbering system, closely paralleling the high non-adoption rates found among interns (79.7%) and clinical students (67.4%). This directly emphasizing the gap that the proposed “qpdb” modification aims to bridge.

**Table 2:** Chi-Square analysis of utilization of tooth numbering systems for supernumerary teeth across different professions.

Professions	Use any tooth numbering system to indicate supernumerary teeth? n (%)		p-value
	Yes	No	
Clinical student	29 (32.6%)	60 (67.4%)	0.057
Intern	12 (20.3%)	47 (79.7%)	
Dentist	34 (39.1%)	53 (60.9%)	
Total	75 (31.9%)	160 (68.1%)	

*Statistically significant difference at *P* < 0.05.

Combining the results of both statistical tests reveals a clear gap in dental practice. On one hand, facing supernumerary teeth is highly common and increases with experience, as 93.1% of practicing dentists have encountered them (*p* < 0.001). On the other hand, the lack of using a specific naming system is a widespread problem that affects students, interns, and experienced dentists almost equally (*p* = 0.057). This contrast where dentists frequently see these teeth but universally lack a standard system to name them provides a strong, data-backed justification for introducing the modified “qpdb” system.

### Implementation of the new modification to system

The “qpdb” teeth numbering system employs four letters to indicate the four quadrants of the oral cavity and each tooth within a quadrant identified by a number from 1to 8 for permanent teeth or letters A to E for deciduous teeth. The present modification incorporates the digit zero (0) to denote the supernumerary teeth within the existing framework. When paired with the code of the tooth positioned mesially to the supernumerary tooth (identifier tooth), the zero serves to produce a precise, unambiguous designation for the supernumerary tooth in question.

Three distinct situations are addressed by this modification:

### The first scenario: Single supernumerary distal to a normal tooth

In such a case one zero will be added to the code of the mesially identifier tooth (tooth mesial to the supernumerary). For example, when a supernumerary tooth appears distal to the permanent maxillary right central incisor receives the designation “q10” by adding the zero to “q1” (which is the number of right maxillary central in “qpdb” teeth numbering system). To reference both teeth (normal and supernumerary) simultaneously, we can either use the notation “q110” or by putting separator, so notation could also be " q1, q10”. This principle extends to be applied to numbering various other conditions including cases of distomolars (distodens), with the exception of mesiodens situations, which require separate later explanation.

### The second scenario: Multiple supernumerary teeth distal to the normal one

When more than one supernumerary tooth is present distal to the same normal one, additional zero is appended for each successive supernumerary tooth reflecting its ordinal position. Specifically, the first supernumerary receives one zero, the second receives two zeros, and the third receives three zeros and so on. For example, when numbering supernumerary teeth positioned distal to the upper left canine (p3), the first supernumerary is designated (p30), the second (p300), the third (p3000), this scalable approach ensures that each tooth has a unique unambiguous code regardless of the number of supernumerary teeth.

### The third scenario: Mesiodens teeth

Mesiodens are extra teeth positioned at the midline between two adjacent quadrants, and therefore they cannot be assigned to a single quadrant letter as in conventional manner so the notation of these teeth is indicated by inserting the zero between letters indicating the two neighboring quadrants, therefore numbering the maxillary mesiodens is noted as (q0p) and for numbering the mandibular mesiodens it is (d0b).

**Fig. 4:**
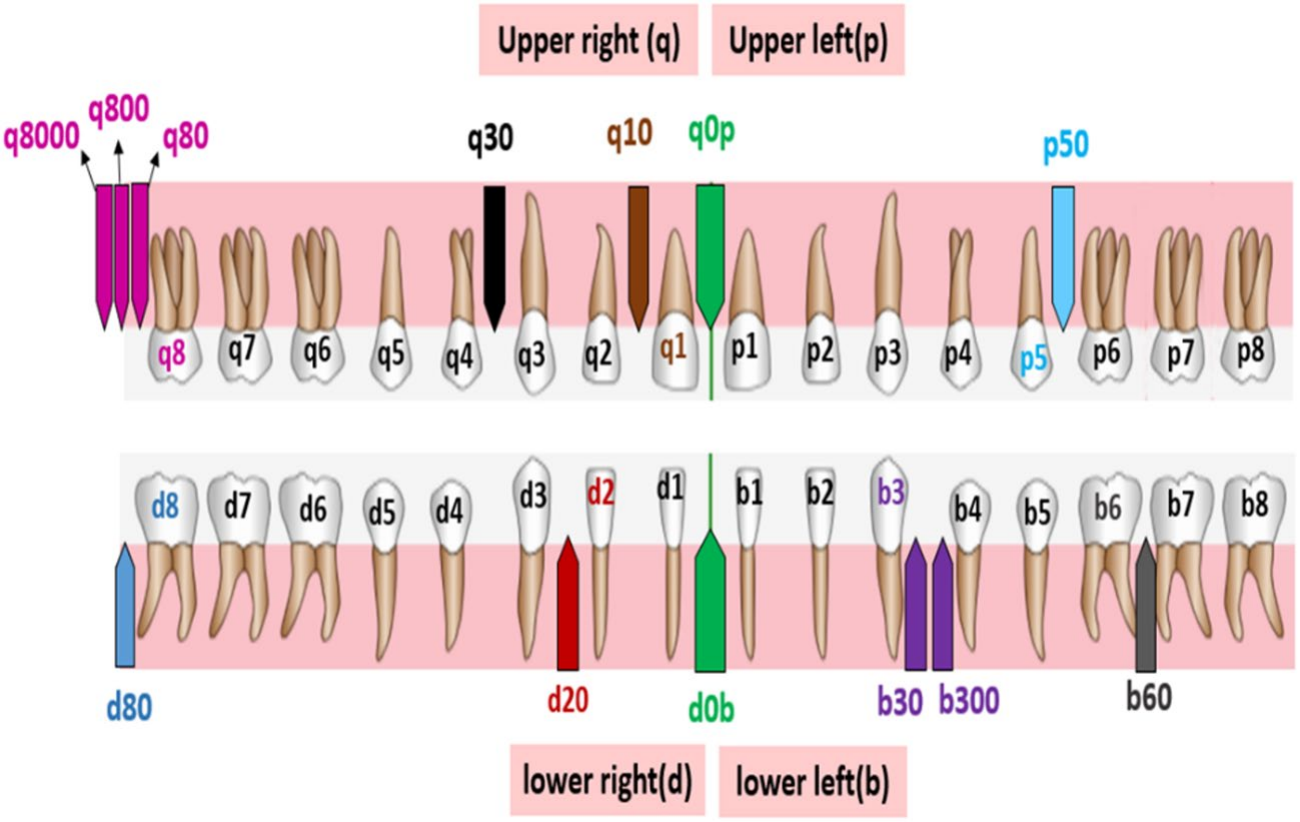
Diagram showing different case scenarios of supernumerary teeth according to the new version of the **(qpdb)** system. **q0p**: upper mesiodens, **d0b**: lower mesiodens, **q10**: supernumerary tooth distal to the upper right central incisor, **q30**: supernumerary tooth distal to the upper right canine, **q80**:first supernumerary tooth distal to the upper right third molar, **q800**: second supernumerary tooth distal to the upper right third molar, **q8000**: third supernumerary tooth distal to the upper right third molar, **p50**: supernumerary tooth distal to the upper left second premolar, **b30**:first supernumerary tooth distal to the lower left canine, **b300**:second supernumerary tooth distal to the lower left canine, **b60**: supernumerary tooth distal to the lower left first molar, **d20**: supernumerary tooth distal to the lower right lateral incisor, **d80**: supernumerary tooth distal to the lower right third molar.

**Fig. 5:**
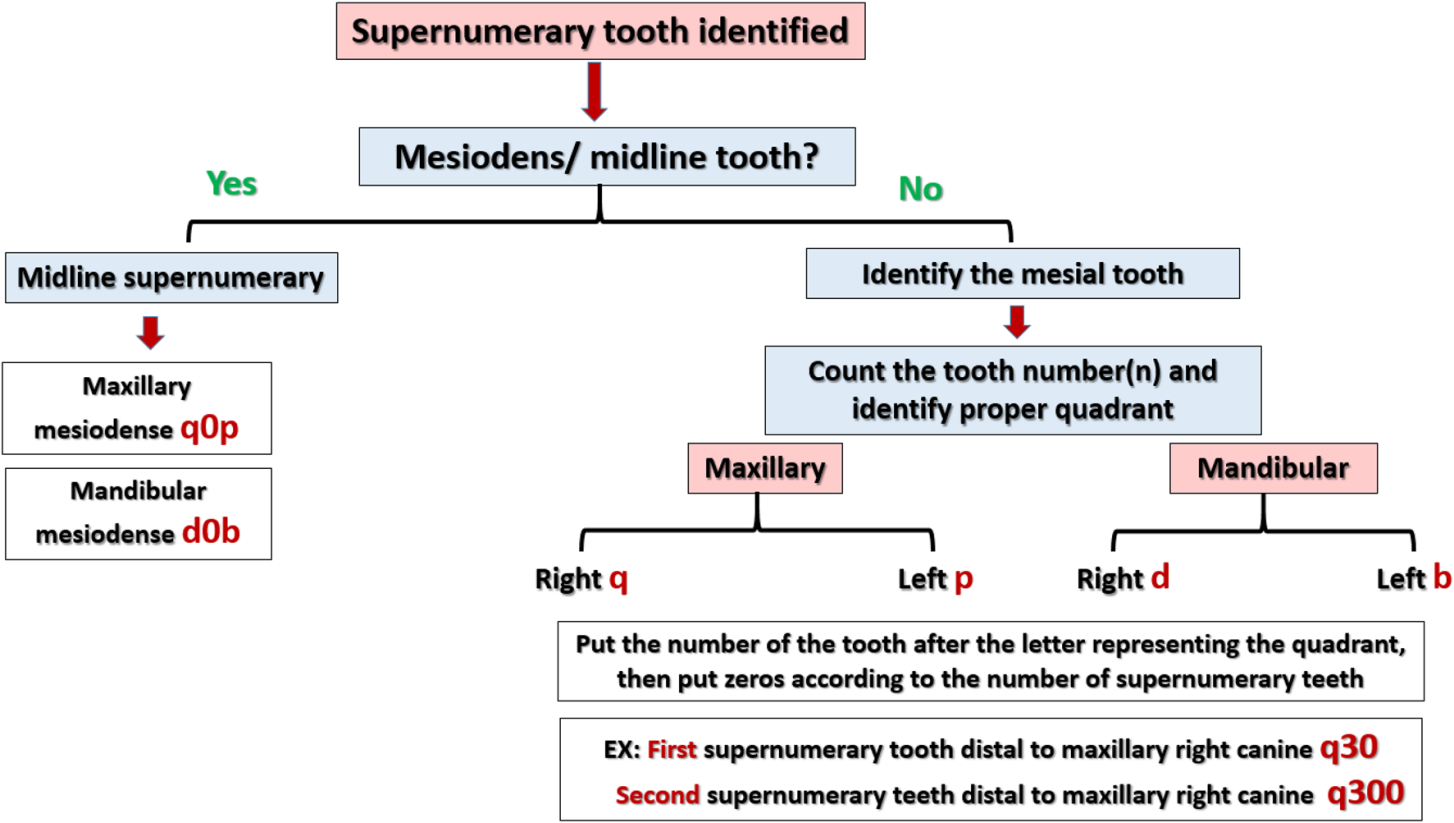
A decision tree flow chart illustrating the step by step application of the modified qpdb numbering system for incorporation of supernumerary teeth.

Morphological descriptors may be added alongside the numeric code using international classification terminology including (conical, tuberculate, supplemental, odontome) as appropriate to the clinical presentation. Examples of the system application were presented in Fig. 4 also a decision tree flow chart illustrating the step by step application and a structured guide illustrating the formula and clinical case scenarios were presented in Fig. 5; Table 3.

**Table 3:**
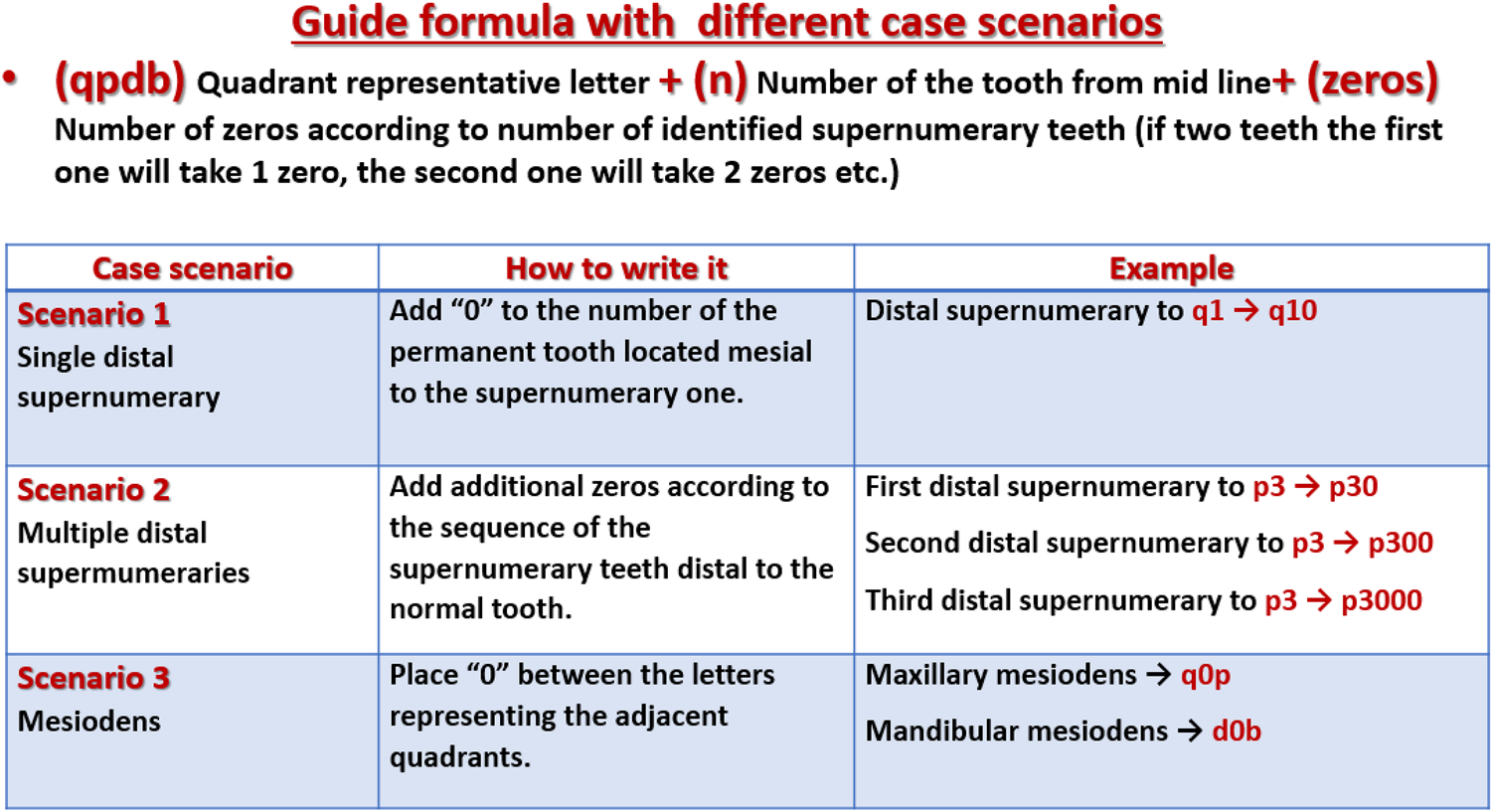
A structured guide illustrating the formula and clinical case scenarios for application of modified qpdb numbering system for supernumerary tooth designation.

## Discussion

This study identified a significant gap in standardized clinical practice, despite 77.4% of surveyed practitioners reporting prior clinical encounters with supernumerary teeth, 68% had no experience with any numbering system for their identification. This finding is consisting with a broader literature acknowledge that existing tooth numbering systems were not designed with supernumerary teeth in mind thus lack the capacity to document them precisely.

The modification in qpdb system directly addresses this gap by introducing a rule based, scalable, and digitally compatible notation method that requires no additional memorization beyond the original qpdb framework. It is a proposed solution designed to address the limitations of existing systems used for teeth numbering.

Supernumerary teeth (SNT) present a unique challenge in dental practice. Accurate identification and documentation of such teeth are essential for diagnosis, treatment planning and inter-professional communication. The three most widely adopted numbering systems, the Universal Numbering System (UNS), the Palmer Notation System and the Fédération Dentaire Internationale (FDI) System each present a distinct limitation when applied to supernumerary teeth documentation^40^.

The Universal Numbering System (UNS), adopted by the American dental association, employs sequential numbering from #1 to #32^41^. To denote SNT, some practitioners append letters (e.g., “a,” “A,” or “S”) to the parent tooth number. However, this can cause confusion, particularly with “S” resembling “5” in written form. The Universal Supernumerary Teeth Numbering System assigns numbers #51– #82 to SNT, offers a more structured alternative however it does not resolve the challenge of multiple SNT adjacent to a single parent tooth and requires practitioners to memorize additional numbers, which limits its practical adoption^40^.

The Palmer Notation System, while effective for quadrant-based classification, does not systematically incorporate SNT. When SNT are present, clinicians must manually annotate the diagram, leading to inconsistencies. Yusof W. used (A, PM & M) for anteriors, premolars and molars respectively to indicate SNT in the regional quadrant Palmer-style notation^42^, while Ferguson J. suggested the use of special symbols for SNT in the Palmer/Zsigmondy Notation System^43^.

Others used the numbers 9, 10, and 11 to denote fourth, fifth, and sixth disto-molars, respectively, but did not have a special designation for a supernumerary premolar^22,44,45^. A further practical limitation of the Palmer system is its reliance on quadrant bracket symbols that are not available on standard keyboards, which significantly complicates its integration into electronic dental records.

The FDI System, a widely accepted international standard, employs a two-digit numbering method for primary and permanent teeth, but like the previous systems, it lacks an intuitive solution for SNT, relying on descriptive notations^41^. Anthonappa et al. appended “ST” (for supernumerary tooth documentation) after the FDI parent tooth number^46^. However, Inchingolo et al. carried on with the FDI system and identified supernumerary fourth and fifth disto-molars in the maxillary left quadrant as “2.9” and “2.10,” respectively^47^. These adaptations while practical within their respective clinical contexts were not standardized or universally adopted, and cannot be considered systematic solution.

Alternative dedicated systems, such as Sarjeev’s supernumerary teeth numbering system, have attempted to integrate SNT into a structured notation. However, these systems often employ complex symbols (e.g., alpha, beta, gamma) and abbreviations (e.g., P for paramolar, DM for distomolar, M for mesiodens), which may complicate clinical documentation and communication^38^.

The new addition to the qpdb numbering system directly addresses these challenges and offers several advantages over the existing approaches. First the use of the digit zero as a supernumerary designator is intuitive and rule based requiring no memorization of new ranges, symbols or abbreviations beyond the qpdb framework. Second, the system is fully scalable, each successive supernumerary tooth in the same region receive additional zero, ensuring that every tooth receives a unique code regardless of the number of the supernumerary teeth present. Third, the mesiodens (the most commonly encountered tooth) has a unique notation which is the first within any establishing notation to have a specific alphanumerical code which is clinically important for this supernumerary type. Fourth, the system uses only standard lowercase letters and numerals ensuring full computability within all electronic dental record platforms without requiring special character encoding, a limitation that affect palmer system particularly.

Morphological classification may similarly be recorded alongside the alphanumeric code, these optional extensions are intentionally designed as supplementary rather than obligatory components, preserving the simplicity of the core system while allowing expanded documentation where clinical need demands it. Table four summaries a comparison between the systems. Table 4.

**Table 4:** Comparison of the qpdb notation with established teeth numbering systems with respect to supernumerary teeth notation^3,38–41,43,46,47^.

Parameter	FDI	Palmer	Universal	qpdb
Supernumerary teeth notation method	✗ No standardized method Some practitioners use “9” as the second digit (e.g., 19 or 29), but this not universally agreed upon. Others rely on free text description.	✗ No standardized method Supernumeraries are typically annotated with an asterisk (*) or described in clinical notes. No consistent alphanumeric convention exists.	✗ No standardized method Some use letter suffixes (e.g., “AS” for anterior supernumerary). Or adding 50 to the number of tooth (e.g., “51”)	**✓** Standardized method is present Zero “0” is appended after the mesially adjacent tooth code to denote a supernumerary.
Mesiodens consideration	✗ No dedicated code exists. Practitioners rely entirely on descriptive text to communicate mesiodense position.	✗ No dedicated code exists. Practitioners rely entirely on descriptive text to communicate mesiodense position.	✗ No dedicated code exists. Practitioners rely entirely on descriptive text to communicate mesiodens position.	**✓** Dedicated notation available q0p = maxillary mesiodens d0b = mandibular mesiodense Position between two quadrants is encoded directly in the notation
Scalability for multiple supernumeraries in the same region	✗ No convention exists for multiple supernumeraries in the same area. Each is described separately in free text.	✗ No convention exists for multiple supernumeraries in the same area. Each is described separately in free text.	✗ No convention exists for multiple supernumeraries in the same area. Each is described separately in free text.	**✓** Each additional supernumerary receives one additional zero: 1 extra tooth → q10 2nd extra → q100 3rd extra → q1000
Ease of learning	► Moderate Requires memorization of the two-digit quadrant-tooth code (quadrants 1–4 for permanent, 5–8 for deciduous). Widely taught in dental curricula internationally.	► Moderate Requires memorization of quadrant bracket/angle symbols and tooth numbers 1–8 for permanent, A-E for deciduous.	**✓** Easy Simple sequential numbering (1–32 permanent, A-T deciduous). Widely taught and familiar in North American dental education.	► Moderate Requires learning the original qpdb quadrant letters (q, p, d, b) and the zero rule for supernumeraries. Intuitive and rule based once the base system is understood
Digital and electronic dental record (EDR) compatibility	**✓** Full compatibility Uses standard numerals only. Compatible with all EDR systems, plain text fields and dental software without special character encoding.	► Partial compatibility Requires quadrant bracket symbols that may not be available on standard keyboards, which complicate digital use.	**✓** Full compatibility Uses standard numerals and letters only. Widely compatible with EDR systems and dental software.	**✓** Full compatibility Uses only standard lowercase letters and numerals. Compatible with all text fields in EDR systems without special encoding or formatting.
Deciduous dentition support	**✓** Yes Uses quadrants 5–8 for primary teeth with the same two-digit format.	**✓** Yes Uses letters A-E for primary teeth in each quadrant.	**✓** Yes Uses letters A-T for the full primary dentition.	**✓** Yes Uses letters A-E for primary teeth in addition with quadrant letter (q, p,d, b).
Current international adoption status	**✓** Widely adopted WHO recommended system. Standard in most of Europe, Asia, Africa and Australia. Used in international dental journals.	► Regional adoption Common in the United Kingdom and some European countries. Less prevalent in international literature.	► Regional adoption Standard in the United States and Canada. Widely taught in North American dental schools.	► Proposed -not yet adopted Proposed modification requiring prospective validation and usability assessment before recommendation for wider clinical adoption.

**Abbreviations**: FDI = Federation Dentaire Internationale; EDR = Electronic Dental Record.

**Symbols**: ✔ Supported/Advantage ✘ Not supported/Limitation ▶ Partial support.

Several limitations of the present study must be acknowledged; the questionnaire was administered using the convenience sampling approaches so the sample size while sufficient for a needs-assessment study, is comparatively modest. The recruitment was conducted within the Egyptian academic networks which may restrict the generalizability of the results, so broader multicenter studies involving practitioners from diverse healthcare settings are recommended to assess the global relevance of the identified need. Future validation study comparing error rates, time taken for identification and user preference are also needed.

Additionally, the suggested qpdb system is innovative and has not yet been adopted in clinical settings, leading to inquiries regarding its practical use in the real world. The current version of the system is also missing a method for indicating the buccolingual position of supernumerary teeth, an essential aspect for thorough documentation.

## Conclusion

The findings of this study highlight the limitations of current dental numbering systems in accurately classifying supernumerary teeth and the potential for the qpdb system to address these shortcomings. By incorporating a clear and consistent method for identifying SNT, the qpdb system can improve communication among dental professionals and enhance the precision of dental records. The use of the digit ‘0’ (zero) in the qpdb system to denote supernumerary teeth simplifies their identification and documentation, offering a more effective alternative to existing systems that rely on ambiguous notations or require memorization of additional numbers. While this study provides a promising foundation for a new numbering system, it is important to acknowledge its limitations, including the sample size and single-site nature of data collection. Further research, utilizing larger and more diverse samples, is recommended to validate the qpdb system and assess its clinical applicability and long-term effectiveness.

## Supplementary Information

Below is the link to the electronic supplementary material.

Supplementary Material 1

Supplementary Material 2

## Data Availability

All data generated or analyzed during this study are included in this article.

## Abbreviations

SNT: Supernumerary teeth
FDI: The fédération dentaire internationale
UNS: Universal numbering system
