# Supplementary material for: Involving supernumerary teeth in “qpdb” teeth numbering system
Source: Sci Rep. 2026 Jul 3;16:20527. doi: 10.1038/s41598-026-58563-2 (PMC13332233; doi:10.1038/s41598-026-58563-2)
Supplement: Supplementary file 1 — Supplementary Material 1 [file 41598_2026_58563_MOESM1_ESM.pdf]

Questionnaire

This survey is completely anonymous, it for research purposes only,Your responses will be published. If you do not wish to have your answers published, please do not respond.

* Indicates required question

1. I confirm that I understand the purpose of the questionnaire and I consent to voluntarily participate and to the publication of results *

   Mark only one oval.

   - Yes
   - No

2. profession *

   Mark only one oval.

   - clinical student
   - intern
   - dentist

3. Have you ever seen a patient with supernumerary teeth *

   Mark only one oval.

   - Yes
   - No

4. How many cases of supernumeraries did you see *

   ____

5. Do you use any tooth numbering system to indicate supernumerary teeth *

Mark only one oval.

- ( ) Yes
- ( ) No

6. What is the most frequent type of supernumerary teeth through your clinical experience *

Mark only one oval.

- ( ) Mesiodens
- ( ) Parapremolars
- ( ) Paramolars
- ( ) Distomolars
- ( ) Other

7. How can you indicate supernumeraries in communication with other dental professions *

This content is neither created nor endorsed by Google.

Google Forms
