# Supplementary material for: Involving supernumerary teeth in “qpdb” teeth numbering system
Source: Sci Rep. 2026 Jul 3;16:20527. doi: 10.1038/s41598-026-58563-2 (PMC13332233; doi:10.1038/s41598-026-58563-2)
Supplement: Supplementary file 2 — Supplementary Material 2 [file 41598_2026_58563_MOESM2_ESM.docx]

| **Profession * Ever_seen Crosstabulation** | | | | | | | | |  |
| --- | --- | --- | --- | --- | --- | --- | --- | --- | --- |
|  | | | Ever_seen | | | | Total | |  |
|  |  |  | Yes | | No | |  |  |  |
| Profession | Clinical student | Count | 54 | | 35 | | 89 | |  |
|  |  | % within Profession | 60.7% | | 39.3% | | 100.0% | |  |
|  |  | % within Ever_seen | 29.7% | | 66.0% | | 37.9% | |  |
|  | Dentist | Count | 81 | | 6 | | 87 | |  |
|  |  | % within Profession | 93.1% | | 6.9% | | 100.0% | |  |
|  |  | % within Ever_seen | 44.5% | | 11.3% | | 37.0% | |  |
|  | Intern | Count | 47 | | 12 | | 59 | |  |
|  |  | % within Profession | 79.7% | | 20.3% | | 100.0% | |  |
|  |  | % within Ever_seen | 25.8% | | 22.6% | | 25.1% | |  |
| Total | | Count | 182 | | 53 | | 235 | |  |
|  |  | % within Profession | 77.4% | | 22.6% | | 100.0% | |  |
|  |  | % within Ever_seen | 100.0% | | 100.0% | | 100.0% | |  |
| **Profession * Use_numbering Crosstabulation** | | | | | | | | | |
|  | | | | Use_numbering | | | | Total | |
|  |  |  |  | Yes | | No | |  |  |
| Profession | Clinical student | Count | | 29 | | 60 | | 89 | |
|  |  | % within Profession | | 32.6% | | 67.4% | | 100.0% | |
|  |  | % within Use_numbering | | 38.7% | | 37.5% | | 37.9% | |
|  | Dentist | Count | | 34 | | 53 | | 87 | |
|  |  | % within Profession | | 39.1% | | 60.9% | | 100.0% | |
|  |  | % within Use_numbering | | 45.3% | | 33.1% | | 37.0% | |
|  | Intern | Count | | 12 | | 47 | | 59 | |
|  |  | % within Profession | | 20.3% | | 79.7% | | 100.0% | |
|  |  | % within Use_numbering | | 16.0% | | 29.4% | | 25.1% | |
| Total | | Count | | 75 | | 160 | | 235 | |
|  |  | % within Profession | | 31.9% | | 68.1% | | 100.0% | |
|  |  | % within Use_numbering | | 100.0% | | 100.0% | | 100.0% | |
